# Effects of Qishe Pill, a compound traditional Chinese herbal medicine, on cervical radiculopathy: study protocol for a randomized controlled trial

**DOI:** 10.1186/1745-6215-14-322

**Published:** 2013-10-07

**Authors:** Xue-Jun Cui, Yue-Li Sun, Sheng-Fu You, Wen Mo, Sheng Lu, Qi Shi, Yong-Jun Wang

**Affiliations:** 1 Longhua Hospital, Shanghai University of Traditional Chinese Medicine, Shanghai 200032, P.R. China; 2 Spine Institute, Shanghai University of Traditional Chinese Medicine, Shanghai 200032, P.R. China

**Keywords:** Cervical radiculopathy, Neck pain, Qishe pill, Traditional Chinese medicine

## Abstract

**Background:**

Neck pain is a common symptom in most patients suffering from cervical radiculopathy. However, some conservative treatments are limited by their modest effectiveness. On the other hand, surgical intervention for cervical disc disorders is indicated when symptoms are refractory to conservative treatments and neurological symptoms are progressive. Many patients use complementary and alternative medicine, including traditional Chinese medicine, to address their symptoms. The purpose of the present study is to examine the efficacy and safety of Qishe Pill, a compound traditional Chinese herbal medicine, for neck pain in patients with cervical radiculopathy.

**Methods/design:**

A multicenter, double-blind, randomized, placebo-controlled trial to evaluate the efficacy and safety of the Qishe Pill is proposed. The study will include 240 patients from five sites across China and diagnosed with cervical radiculopathy, according to the following inclusion criteria: age 18 to 65 with pain or stiffness in the neck for at least 2 weeks (neck disability index score 25 or more) and accompanying arm pain that radiates distally from the elbow. Qualified participants will be randomly allocated into two groups: Qishe Pill group and placebo group. The prescription of the trial medications (Qishe Pill/placebo) are 3.75 g each twice a day for 28 consecutive days. The primary outcome is pain severity. Secondary outcomes are functional status, patient satisfaction, and adverse events as reported in the trial.

**Discussion:**

Qishe Pill is composed of processed *Radix Astragali*, Muscone, *Szechuan Lovage Rhizome*, *Radix Stephaniae Tetrandrae*, Ovientvine, and *Calculus Bovis Artifactus.* According to modern research and preparation standards, Qishe Pill is developed to improve on the various symptoms of cervical radiculopathy, especially for neck pain. As it has a potential benefit in treating patients with neck pain, we designed a double-blind, prospective, randomized-controlled trial and would like to publish the results and conclusions later. If Qishe Pill can alleviate neck pain without adverse effects, it may be a unique strategy for the treatment of cervical radiculopathy.

**Trial registration:**

This study is registered at ClinicalTrials.gov, NCT01274936

## Background

Cervical radiculopathy is a distinct consideration in the evaluation of any patients who have neck pain and may be defined simply as an abnormality of a nerve root, which originates in the cervical spine [1]. The typical cervical radiculopathy patient presents neck and arm discomfort of insidious onset. The discomfort can range from a dull ache to a severe burning pain. Typically, pain is referred to the medial border of the scapula, and the patient’s chief complaint is shoulder pain. As the radiculopathy progresses, the pain radiates to the upper or lower arm and into the hand, along the sensory distribution of the nerve root that is involved. The initial approach to the management of cervical spondylopathic radiculopathy is nearly the same as that of non-specific neck or back pain that can be found in most patients. Conservative treatments include non-steroidal anti-inflammatory drugs, narcotics, muscle relaxants, physical therapy, and transcutaneous electrical nerve stimulation. The main objectives of conservative treatments are to relieve pain and improve function and health-related quality of life [2]. However, these treatments for cervical radiculopathy are limited by their modest effectiveness [2]. Surgical treatment for cervical disc disease is indicated when symptoms are refractory to conservative treatments and neurological symptoms are progressive [3]. In terms of pharmacotherapy, there is generally no randomized, placebo-controlled trial available comparing standard non-surgical treatment [4]. Therefore, care plans should be designed based principally on accumulated experience, locally available services, and patient preferences. Treatment plans are developed to alleviate pain, improve function, and prevent recurrence [2].

As a complementary and alternative medicine, herbal medicines have the potential to avoid the adverse effects of medications and surgery [4]. Natural substances, including herbal medicines, have been used to promote healing and alleviate neck pain in western countries [5]. Previous studies have demonstrated that some active substances in herbal medicine can promote Qi flow and blood circulation to alleviate pain.

A number of studies on the effects of the Chinese herbal medicine on cervical radiculopathy have been proposed, but useful empirical research is insufficient. For chronic neck pain, with or without radicular symptoms, there is low quality evidence suggesting that herbal medicine is more effective than placebo for pain relief as measured at the end of the treatment [6]. Moreover, the size of the studies has been small and the effect was measured only in the short-term. Further research is very likely to change both the effect size and our confidence in the results. There is a need for randomized-controlled trials (RCTs) with adequate numbers of participants that address long-term efficacy of herbal medicines compared to placebo.

Using a well-designed clinical trial, we will survey the efficacy of concurrent use of this remedy in relieving neck pain. Therefore, the present study is designed to examine efficacy and safety of Qishe Pill, a traditional Chinese herbal medicine (TCM) compound, on neck pain in cervical spondylopathic radiculopathy in a randomized, double-blind, placebo-controlled trial. Results of this study will provide evidence regarding the value of Qishe Pill as an intervention to alleviate neck pain caused by cervical radiculopathy.

### Details of intervention

Qishe Pill is composed of processed *Radix Astragali*, Muscone*, Szechuan Lovage Rhizome*, *Radix Stephaniae Tetrandrae*, Ovientvine*,* and *Calculus Bovis Artifactus* (Table 1) [7].

**Table 1:** Standard formula (Capsule ingredients)*

**Chinese name**	**Pharmaceutical name**	**Powered herb,%**
Huang Qi	*Radix Astragali*	28%(13 g)
She Xiang	Muscone	0.06%(0.03 g)
Chuan Xiong	*Szechuan Lovage Rhizome*	26%(12 g)
Fang Ji	*Radix Stephaniae Tetrandrae*	19%(9 g)
Qing Feng Teng	Ovientvine	26%(12 g)
Niu Huang	*Calculus Bovis Artifactus*	0.6%(0.3 g)

*Pharmaceutical Terminology from Hsu [7].

Qishe Pill is a thin 0.15 g film-coated pill; removal of the film reveals alight brown to brown colour. The pill has a slight odor and tastes bitter. The dosage is 25 pills, twice daily, with a total daily dosage of 7.5 g, which is in the dosage range defined by the National Center for Complementary and Alternative Medicine: *Astragalus mongholicus* 9–30 g; *Rhizoma ligustici wallichii* 3–9 g; *Radix Stephaniae Tetrandrae* 4.5–9 g; *Caulis Sinomenii* 6–12 g; Muscone 0.03–0.1 g; *Calculus Bovis Artifactus* 0.15– 0.35 g.

The production process of Qishe Pill: (1) Volatile oil is extracted from *Rhizoma ligustici wallichii*. (2) The remaining drugs forma decoction is mixed with *astragalus membranaceus* soaked for 30 minutes and simmered in 10 times the amount of water, twice, for 2 hours each time. (3) The decoction is then vacuum filtrated to a concentration of relative density 1.24-1.26 (70°C). Ethanol is mixed into the concentrated decoction to reach 70% alcohol content. (4) After standing, filtering, and concentrating with recovery of ethanol, the decoction is crushed into a fine powder after vacuum drying. (5) In the reagent of 14 times the amount of 70% ethanol, *Radix Stephaniae Tetrandrae* and *Caulis Sinomenii*are is extracted with the circumfluent alcohol three times, once 2 hour. (6) Volatile oil of *Rhizoma ligustici wallichii* is mixed with 4 times the amount of β-cyclodextrin and then dried in vacuum. (7) The porphyrization of *Artificial Calculus Bovis Artifactus* and *Artificial Muscone* is mixed with β-cyclodextrin and other accessories to be made into a piece, pelleted and coated pills. These pills are called Qishe Pill in drug name.

The placebo is composed of β-cyclodextrin, colorants and food flavoring, to look and taste similar to Qishe Pill.

## Methods/design

### Study design

This clinical trial is a multi-center, randomized, double-blind, placebo-controlled design. Participants will be enrolled by clinic recruitment at five hospital sites (Longhua Hospital, Shanghai University of TCM; Huadong Hospital, Fudan University; Affiliated Hospital of Changchun University of TCM; TCM Hospital of Gansu province; and TCM Hospital of Suzhou city). Participants will be recruited in equal numbers (n=48) from each of the five sites.

### Ethical issues

This study has been approved by the Institutional Review Board of Longhua Hospital, Shanghai University of TCM on November 30^th^ 2011 (No: 2011LCSY031). All study participants will sign the written informed consent prior to participation. All patients will receive written and oral reassurance regarding the typical benign course of the symptoms. We will explain that placebo and treatment with Qishe Pill might be equally effective interventions. Patients in all treatment groups will be allowed to use painkillers when necessary. In general, paracetamol is selected with or without a non-steroidal anti-inflammatory drug. Patients will be asked to note their drug use, including over-the-counter analgesics, in a specially designed diary during the first four weeks following randomization.

### Patient population and recruitment procedure

All participants will be selected from the general outpatient clinic offive hospital sites, where the patients willbe diagnosed with cervical radiculopathy confirmed by a neurologist, and then diagnosed based upon clinical manifestations (pain along the cutaneous distribution of one or more cervical roots, which may include weakness and hyporeflexia), physical examination, and imaging [4].

Inclusion criteria are age between 18 and 65 years, pain or stiffness in the neck for at least 2 weeks, neck symptoms reproducible during physical examination, neck pain on neck disability index (NDI) of 18 or more, and radiation of arm pain distal to the elbow, plus at least one of provocation of neck or arm pain by neck movements, Brachial plexus traction test, foraminal compression test, or foraminal separation test, sensory changes in one or more adjacent dermatomes, or muscle weakness in one or more adjacent myotomes. Further inclusion prerequisites are willingness for treatment and to adhere to measurement regimens, no involvement in litigation, and written informed consent.

Excluded criteria are patients whose history, signs, and symptoms suggested a potential non-benign cause (including previous neck surgery) or evidence of a specific pathologic condition, such as malignancy, neurologic disease, fracture, herniated disc, or systemic rheumatic disease, clinical signs of spinal cord compression, previous neck trauma or treatment with surgery, obvious vertigo, pregnant women, lactating women, currently participating in other clinical trials, accompanied with hepatic, renal, hematopoietic system, endocrine system, cardiovascular, nervous system, primary and other serious diseases (serum ALT, AST, ALP, TBIL, DBIL, Cr or BUN more than two-fold than the upper limit of reference value), tuberculosis, vertebral deformities, cancer and mental illness, and insufficient understanding of the Chinese language, and/or have been treated with physical therapy or manual therapy for neck pain during the previous 2 weeks.

Patients with concurrent headaches, non-radicular pain in the upper extremities, and low back pain will not be excluded, but neck pain has to be the main symptom for all patients [8].

Patients will be recruited from May 2012 to Oct 2013 from the five sites in China. The review board at each institution approved the protocol. The study is organized and coordinated by Longhua Hospital, Shanghai University of TCM in Shanghai, China.

This study is to be conducted according to the protection of patients, as outlined in the Declaration of Helsinki, and approved by the appropriate Institutional Review Boards. Each participant will sign the written informed consent before undergoing any examination or study procedure, in compliance with Good Clinical Practice. Patients who initially meet these eligibility criteria are then to complete the additional baseline testing and will be randomized into either the treatment or the control group. An independent steering committee will oversee the study.

### Interventions

At randomization, patients will be assigned to receive Qishe Pill (or matching placebo) at 3.75 g twice per day. In this study, the same outer packing will be used for both Qishe Pill and the matching placebo. Therefore, the treating physicians, participants and investigators will be blinded to treatment assignment. The Qishe Pill and matching placebo used in the trial are both manufactured and provided by a pharmaceutical company that meets the requirements of Good Manufacturing Practice. All significant medicine information, including ingredient composition, heavy metals, etc., is provided by the same company.

Follow-up visits will be continued for 5 months. At each visit, data will be collected on the outcome events, compliance and adverse reactions leading to a discontinuation of study medications. All primary and secondary events will be documented and be centrally adjudicated with the use of standardized definitions.

### Randomization and allocation

Treatment allocation occurs when the study participant meets the inclusion criteria and signs the informed consent form. All participating patients will be randomized using a computerized block randomization schedule with a multiple block size of four; 240 patients will be randomly generated and kept in sequentially numbered opaque envelopes. All of the Qishe Pill and placebo provided by the pharmaceutical company will be numbered with a label according to the randomization schedule. When researchers administer the intervention medicine (Qishe Pill or matching placebo), these should be administered according to the randomized number. Randomization and uncovering must be conducted by a statistician team as a third party. As a result, researchers and patients are totally blinded to treatment allocation; the only un-blinded individual is the statistician responsible for the randomization process. The treatment assignments are balanced within each block.

### Outcome measurements

The primary study outcome is pain severity (measured with a visual analogue scale (VAS)). Secondary outcomes are a composite of functional status (measured by NDI), patient satisfaction, and adverse events as reported in the trial. The VAS measures the amount of pain, which is a pain score ranging from 0 (no pain) to 100 mm (very severe pain) [9]. Operationally, the VAS score is displayed as a horizontal line, 100 mm in length, with word descriptors anchored at each end. The patient marks on the line the point that they feel represents their perception of their current pain. The VAS score is then determined by measuring in millimeters from the left hand end of the line to the point that the patient marks. The VAS score and NDI will be measured at all the measurement points (baseline, 2 and 4 weeks of treatment duration, and 2 and 5 months of follow-up duration).

### Study schedule

The measurements that need to be performed at each visit are listed in Table 2.

**Table 2:** Study schedule of clinical trial (6 months)

	**Initial screening**		**Treatment times**		**Follow-up**	
	**Visit 1**	**Visit 2**	**Visit 3**	**Visit 4**	**Visit 5**	**Visit 6**
	**Week 0**	**Week 1**	**Week 2**	**Week 4**	**Month 2**	**Month 5**
Determined the exclusion criteria	Yes					
Informed consent	Yes					
Randomized	Yes					
Weight	Yes		Yes	Yes		
Blood pressure	Yes		Yes	Yes		
Heart rate/Rhythm	Yes		Yes	Yes		
Respiratory rate	Yes		Yes	Yes		
Spurling’s test	Yes					
Brachial plexus force test	Yes					
Drug allergy history	Yes					
Blood routine	Yes			Yes		
Urine routine	Yes			Yes		
Excrement routine	Yes			Yes		
Occult blood	Yes			Yes		
ECG	Yes			Yes		
Liver function test	Yes			Yes		
Kidney function test	Yes			Yes		
X-ray (frontal and lateral)	Yes			Yes		
Concomitant medication		Yes	Yes	Yes		
VAS scores	Yes	Yes	Yes	Yes	Yes	Yes
NDI scores	Yes		Yes	Yes	Yes	Yes
SF-36 scores		Yes	Yes	Yes		
The medicine issue		Yes	Yes			
Compliance assessment			Yes	Yes		
Degree of satisfaction				Yes		
Safety evaluation		Yes		Yes		

ECG: Electrocardiogram; VAS: Visual analogue scale; NDI: Neck disability index: SF-36: The MOS item short from health survey.

### Assessment of adverse events

All subjects are to be questioned regarding adverse events during treatment at each visit; all reported adverse events will be analyzed in spite of the investigators’ assessments of causality and will be categorized according to the Medical Dictionary for Regulatory Activities (MedDRA, Version 8.1J).

### Sample size considerations

We calculated the sample size for this two arm trial on the basis of the comparison treatment (Qishe Pill) versus placebo, with equal allocation in the treatment arms and five repeated measurements (at entry, at 2 and 4 weeks’ treatment, and at 2 and 5 months’ follow-up), with an estimated correlation coefficient of the measurements of ρ=0.8 and a difference in the mean value of the VAS for neck pain of 12 mm, as a clinical relevant difference with an estimated standard deviation in each treatment group of 25 mm [10]. As neck pain is the main complaint in cervical radiculopathy, we chose this outcome to calculate the sample size. Under the setting of alpha = 0.05 and power = 90%, the calculated sample size of each group is around 96. Considering 20% loss to follow-up, the total sample size needed to detect this difference at a 5% level of significance with a power of 90% is 240 patients.

### Statistical analysis

The data will be collected and analyzed according to the intention-to-treat principle. Standard statistical techniques will be used to describe characteristics of patients in both groups. We will compare baseline characteristics in both groups and if incomparability appears, we will perform the secondary analysis, adjusting for differences. The primary outcome, amount of pain, will be compared between both groups using survival analysis and analysis of variance for repeated measures. If adjustment for possible baseline incomparability is needed, analysis of covariance will be performed. We will use multivariate analysis to do retrospective analysis with the baseline data of the effective cases and ineffective cases in the treatment group. Differences in the baseline data will be used to optimize the inclusion criteria. We will do subgroup analysis based on severity of pain (VAS=70 mm) and use of analgesics.

## Discussion

Cervical radiculopathy is a significant public health problem worldwide, accounting for 60% to 70% of all cases of cervical spondylopathy; it is generally believed that most symptoms can be cured or relieved by non-surgical treatment [11]. The chief symptom of cervical radiculopathy is neck pain, which usually originates from a variety of non-specific musculoskeletal causes, including direct trauma, progressive structural changes with or without associated systemic disease (e.g., rheumatoid arthritis), degenerative conditions, and chronic stress or strain injury [12,13]. A Canadian-based cohort reported that, although cervical spondylopathic radiculopathy is less common among the annual incidence of neck pain (14.6%), a systematic approach to its evaluation when encountered is no less important [13]. Therefore, those considerations are unique to cervical radiculopathy in the assessment of patients who have neck pain and the treatment of neck pain relief.

Analgesic and nutritive nerve medicine have considerable effects on neck pain. Analgesics, such as aspirin, seem to be relatively secure, but it is reported that gastrointestinal reactions often appear [14]. Mecobalamin, a neuropathy drug, can relieve neck pain by means of stimulating the inhibition of nerve degeneration and improving the excitability of nerve fibers [15]. However, it seems to be too late to cure oncenerve degeneration occurs.

Qishe Pill provides specific benefits for “Qi and Blood”, terms in TCM, which can profit and activate blood circulation. Using the model animal pathology comprehensive validation, other effects have been found that indicate involvement in inhibition of disc inflammation factors and delayed disc cell apoptosis [8]. According to modern research and preparation standards, Qishe Pill was developed to improve the various symptoms of cervical radiculopathy, especially for neck pain.

Qishe Pill is composed of processed *Radix Astragali*, *Muscone*, *Szechuan Lovage Rhizome*, *Radix Stephaniae Tetrandrae*, *Ovientvine*, and *Calculus Bovis Artifactus*. Based upon the theory of TCM prescription, there is a leading, coordinating, auxiliary, and guiding effect in the remedy components. In Qishe Pill, *Radix Astragali* and *Szechuan Lovage Rhizome* have leading effects, *Muscone*has a coordinating effect, *Radix Stephaniae Tetrandrae* and *Ovientvine*have auxiliary effects, and *Calculus Bovis Artifactus*has guiding effects. Each component of Qishe Pill is thought to be beneficial to neck pain patients. The ability of AS-IV, which can be found in *Radix Astragali* to inhibit the NF-qB pathway, might be one underlying mechanism contributing to its anti-inflammatory potential *in vivo*[16]. The *Szechuan Lovage Rhizome* extract has been found to have the ability to inhibit platelet surface activity and platelet aggregation [17]. The *Radix Stephaniae Tetrandrae* exerts anti-inflammatory effects by interfering with ROS production and Ca^2+^ influx through G protein modulation to prevent Mac-1 up-regulation in neutrophil activation [18]. Ovientvineis used in TCM to treat pain, arthritis, etc., and its extract could inhibit synthesis and release of local PGE [19]. *Calculus Bovis Artifactus* could decrease the malondialdehydecontent in inflammatory exudates induced by carrageen an in rats [20]. Pretreatment with muscone inhibited the IL-1beta-induced phosphorylation of extracellular signal-regulated kinases 1/2 and c-Jun N-terminal kinase in a dose-dependent manner, and the expression of prostaglandin E2, 6-keto-prostaglandin F1alpha, IL-1beta, and tumor necrosis factor alpha, and recovered the structural distortion of the degenerative disc. Musconeis a promising agent in the treatment of intervertebral disc degeneration through its anti-inflammatory effects [21]. Qishe Pill prevents intervertebral disc degeneration induced by upright posture [22].

Outcome measures assessing patients with neck pain are used widely in research and in clinical settings to establish baselines, to evaluate the effect of an intervention, and to motivate patients to evaluate their treatment. Generally, the VAS measurements have been found to be both valid and reliable. In many studies, the VAS has been shown to be the easiest to use and is considered to provide the most reliable measurements of pain intensity; it is therefore used as the criterion standard against new rating methods [23]. Similarly, the NDI, a neck-specific questionnaire, has been cited in the literature as the criterion standard for many other questionnaires and is the most valid of the tools reported [24-27]. Furthermore, the NDI discriminates between those who improved or deteriorated and, as expected, does not detect change in score in those who remained stable [28]. Therefore, for the self-effectiveness assessment, we selected the VAS for self-assessment of pain and NDI for self-assessment of function.

We intend to conduct this well designed RCT to investigate both the efficacy and safety of Qishe Pill in patients with neck pain, and consider that conscientiously performed studies and positive outcomes can benefit patients with neck pain. If this study demonstrates the efficacy and safety of Qishe Pill in significant strides, it will contribute to a useful clinical therapy for relieving neck pain in cervical radiculopathy.

## Trial status

Participant recruitment began in September 2012 and finished in three sites (Longhua Hospital, Shanghai University of TCM; Affiliated Hospital of Changchun University of TCM; TCM Hospital of Gansu province); while 28 participants have been enrolled in Huadong Hospital, Fudan University and 30 in TCM Hospital of Suzhou city.

We periodically organized meetings to give participants feedback(recruitment or follow-up) on progress and conducted regular quality controls of all the trial sites by means of telephone conversations with every participant to verify the case report form. Data collection will be recorded in an eCRF system during the clinical trial.

## Abbreviations

NDI: Neck disability index; RCT: Randomized Controlled Trial; TCM: Traditional Chinese medicine; VAS: Visual analogue scale.

## Competing interests

The authors declare that they have no competing financial interests. We gratefully acknowledge the assistance of Michael R. Berger, M.S. Medicine-Acupuncture, Tuina, in revising the manuscript.

## Authors’ contributions

XJC participated in the study design and critical revision of the manuscript. YLS drafted the manuscript. SFY participated in statistical design. WM and SL participated in the study design. QS formulated Qishe Pill. YJW was the general supervisor for this research and participated in both the study design and in the critical revision of the manuscript. All authors read and approved the final manuscript.
